# Optimal Recipient Nerve Selection for Breast Neurotization with Abdominal Flaps: A Comprehensive Meta-Analysis of Anterior and Lateral Intercostal Approaches

**DOI:** 10.3390/jcm14155461

**Published:** 2025-08-03

**Authors:** Woonhyeok Jeong, Jaehoon Choi, Junhyung Kim, Daegu Son, Taehee Jo

**Affiliations:** Department of Plastic and Reconstructive Surgery, Dongsan Hospital, Keimyung University School of Medicine, Daegu 42601, Republic of Korea

**Keywords:** breast reconstruction, neurotization, anterior cutaneous branch, lateral cutaneous branch, sensory restoration

## Abstract

**Background:** Breast reconstruction post-mastectomy has increasingly emphasized the importance of sensory restoration. This study aimed to evaluate the comparative efficacy of anterior versus lateral cutaneous intercostal nerve branches in neurotization during abdominal-based autologous breast reconstruction. **Methods:** Through a systematic literature search and meta-analysis, we reviewed studies published between January 2003 and August 2023. Our methods involved categorizing studies based on the nerve branch used, extracting relevant data, and conducting a quality assessment. To determine the difference in the magnitude of sensory recovery, a meta-analysis was conducted to pool the effect sizes (mean differences) from individual studies. Given the potential for heterogeneity across studies, a random-effects model was employed using the DerSimonian and Laird method. Subgroup analysis was then performed to separately evaluate the effect sizes for the anterior and lateral groups. **Results:** We identified five studies for the anterior group and five studies for the lateral group. The anterior group included a total of 225 non-neurotized and 240 neurotized breasts, while the lateral group consisted of 62 non-neurotized and 51 neurotized breasts. The anterior group exhibited superior sensory recovery compared to the lateral group (*p* = 0.08 for the common effect model). The result was borderline significant, suggesting a trend towards a difference between the two groups. In terms of patient-reported outcomes, the anterior group provided data, while the lateral group lacked such data, underscoring a potential research gap. **Conclusions:** Results indicated a trend favoring the anterior cutaneous branch, with studies showing improved sensory outcomes and patient satisfaction. However, the choice between the two should be individualized, considering the patient’s unique needs and the surgeon’s expertise.

## 1. Introduction

Breast reconstruction following mastectomy has evolved substantially over recent decades, incorporating both implant-based and autologous approaches. Implant-based reconstruction, characterized by advancements in mastectomy techniques such as nipple-sparing mastectomy (NSM), has greatly enhanced aesthetic outcomes without compromising oncologic safety [1,2]. Key considerations include mastectomy flap quality, nipple perfusion, and patient-specific factors like BMI and radiation exposure, all of which significantly influence reconstructive outcomes [3,4,5,6]. Modern highly cohesive silicone implants provide improved form stability and reduced rippling, essential for achieving satisfactory aesthetic results, particularly in prepectoral reconstruction settings [7]. Additionally, adjunctive materials like acellular dermal matrix (ADM) have shown advantages in minimizing complications such as capsular contracture, though they also introduce potential risks like infection and seroma formation [8]. Consequently, contemporary implant-based reconstruction is characterized by careful patient selection, advanced surgical techniques, and thoughtful utilization of support materials, which collectively enhance aesthetic and functional outcomes [9,10,11].

In parallel, fat grafting also has emerged as a promising modality in breast reconstruction, garnering increasing attention despite not yet being widely adopted as a standalone approach. Especially with the advent of more refined techniques and the development of objective pre- and postoperative assessment tools, fat grafting is positioned to play a more central role in reconstructive strategies [12,13]. In the context of breast cancer reconstruction, fat grafting is generally considered both efficacious and oncologically safe; however, further data is required to comprehensively assess its long-term safety in both irradiated and non-irradiated mastectomy patients [14]. Exclusive fat grafting often necessitates multiple sessions to achieve satisfactory outcomes, particularly in irradiated fields. Beyond its oncological safety, fat grafting offers aesthetic benefits, such as improved contour and symmetry, and can address complications commonly associated with implants [15]. Systematic reviews and meta-analyses to date have demonstrated no significant increase in local–regional recurrence or adverse effects on disease-free survival, further supporting its growing role in reconstructive practice [16,17,18,19].

Autologous abdominal-based reconstruction has also undergone substantial refinement. Techniques utilizing the deep inferior epigastric perforator (DIEP) flap are now preferred due to their muscle-sparing design, which minimizes donor-site morbidity while preserving excellent aesthetic outcomes [20]. These procedures depend heavily on detailed preoperative imaging and meticulous microsurgical execution, both of which contribute to reduced complication rates and operative times. More recently, innovations such as stacked or hybrid flaps, including combinations of DIEP and deep circumflex iliac artery (DCIA) flaps, have expanded the reconstructive toolbox, allowing surgeons to address patients with limited donor tissue or complex defect requirements [21]. Thus, abdominal-based reconstruction remains a gold standard, continuously refined through detailed anatomical knowledge, improved surgical approaches, and advances in flap selection and utilization [22].

One area of growing interest in the field of post-mastectomy reconstruction is the restoration of breast sensation, which has a significant impact on patient satisfaction and quality of life [23]. Neurotization, the surgical coaptation of donor and recipient sensory nerves, offers a promising strategy to reestablish tactile and erogenous sensibility, particularly in the nipple–areola complex (NAC) [24]. Although currently underutilized, neurotization has demonstrated both functional and patient-reported benefits with minimal added morbidity [25,26]. In autologous reconstruction, especially with abdominally based flaps, neurotization is facilitated by the availability of donor sensory nerves. This approach has been associated with earlier and more robust sensory recovery compared to non-innervated flaps [27,28]. Conversely, in implant-based reconstruction, where donor nerves are absent, nerve allografts are used to bridge recipient intercostal nerves to retroareolar targets [29]. While technically more limited, recent studies have shown meaningful sensory restoration even in prepectoral, direct-to-implant settings, particularly following nipple-sparing mastectomy. As neurotization techniques continue to evolve, a comprehensive understanding of their anatomical and reconstructive implications is essential for optimizing reconstructive outcomes.

In abdominally based flap neurotization, a pivotal component of this sensory restoration is the selection of the appropriate recipient nerve. The breast is primarily supplied by branches of the intercostal nerves. From the lateral side, the lateral cutaneous branches of the second to the sixth intercostal nerves are involved. Medially, the breast is supplied by the anterior cutaneous branches of the second to sixth intercostal nerves [30]. Consequently, these two nerve branches have emerged as the predominant choices for sensory restoration in this field [31,32]. Each branch has unique anatomical trajectories and implications for surgical outcomes.

The anterior cutaneous branch, coursing anteriorly and medially, has been lauded for its proximity to the primary surgical site in many breast reconstructions [33]. Conversely, the lateral cutaneous branch, with its lateral trajectory, offers a different set of advantages and challenges [34]. The choice between these two branches is not merely anatomical but has profound implications for postoperative sensory outcomes, patient satisfaction, and overall quality of life [35].

Despite the growing body of literature on this subject, a comprehensive understanding of the comparative benefits and potential drawbacks of these two nerve choices remains elusive [36]. High-impact studies have often focused on individual surgical techniques or specific patient outcomes, leaving a gap in the holistic understanding of the broader implications of nerve selection.

This meta-analysis seeks to bridge this knowledge gap, providing a comprehensive review of the current literature on the anterior and lateral cutaneous branches of the intercostal nerves as recipient nerves in breast reconstruction. By synthesizing the findings from diverse studies, we aim to offer clear guidance for surgeons and underscore the significance of informed nerve selection in optimizing patient outcomes.

## 2. Materials and Methods

A systematic review and meta-analysis were conducted following the PRISMA (Preferred Reported Items for Systematic Review and Meta-analysis) guidelines and AMSTAR (Assessing the Methodological Quality of Systematic Reviews) checklist [37,38]. The literature search was conducted by two independent reviewers using the PubMed database. This study was conducted following systematic review methodology; however, it was not pre-registered due to its evolution from an initial narrative overview to a more structured systematic approach. The search was confined to articles published from January 2003 to August 2023. The following search terms were employed: “breast neurotization”, “breast reinnervation”, “breast nerve coaptation” and “innervated breast flap”. From the initial search, a total of 346 articles were identified. After removing duplicates and irrelevant articles, 207 articles were selected for abstract review.

Following a thorough abstract review, 18 articles were deemed relevant for a full-text review. At this stage, 149 studies were excluded due to lack of relevance to breast neurotization procedures, 6 were cadaveric studies without clinical outcomes, 9 were commentary or opinion pieces, 13 were review articles, and 12 were not written in English. Of the 18 full-text articles reviewed, 4 were excluded because they focused solely on allograft- or conduit-based nerve reconstruction without direct neurorrhaphy. An additional 3 studies were excluded for utilizing axillary or unspecified recipient nerves, which could not be classified as anterior or lateral intercostal branches. One study was excluded due to the absence of an appropriate control group for comparison.

**Figure 1:**
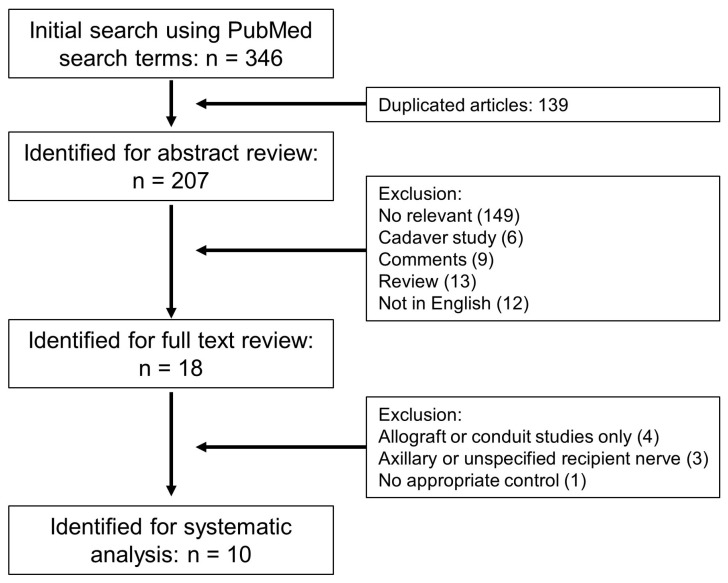
Systematic literature search and exclusion process.

Due to the abundance of non-randomized studies of interventions (NRSIs) in our research topic, as identified in our initial literature review, we decided to focus exclusively on these studies for our investigation. For our analysis, we exclusively selected cases involving direct neurorrhaphy, excluding studies that primarily employed allografts or nerve conduits. From this refined list, 10 met our inclusion criteria and were incorporated into the systematic analysis (Figure 1) [27,33,34,39,40,41,42,43,44,45].

For each of the selected studies, the following data were extracted: study design and level of evidence, patient demographics, breast reconstruction type, number of participants or breasts reconstructed, methodologies used for sensory testing, patient-reported outcomes and satisfaction scores, and complication rates associated with neurotization. Studies were categorized into the anterior group if they used the anterior cutaneous branch of the intercostal nerve as the recipient nerve. Conversely, the lateral group consisted of studies that selected the lateral cutaneous branch of the intercostal nerve as recipients.

**Figure 2:**
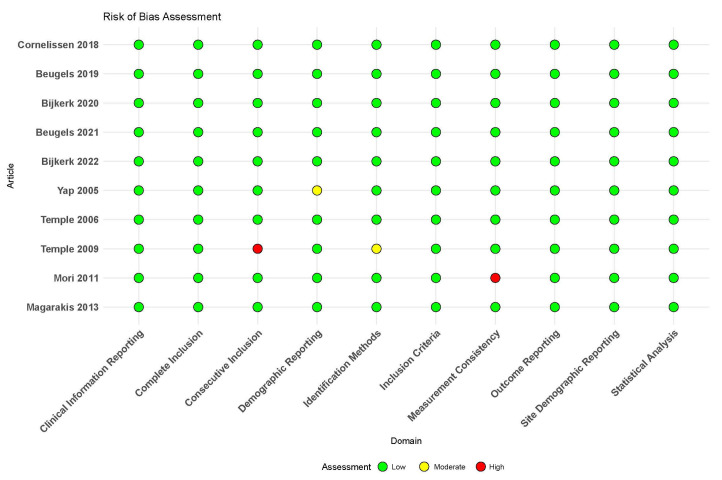
Traffic light plot for risk of bias in individual studies [27,33,34,39,40,41,42,43,44,45].

To ensure a thorough and standardized evaluation of study quality, we employed the Joanna Briggs Institute (JBI) critical appraisal checklist for case series [46]. This comprehensive tool enabled us to assess each study against established inclusion criteria, measurement consistency, identification methods, and additional domains critical to establishing robustness and reliability. The outcomes of this appraisal were then succinctly visualized using a traffic light plot, which provided a clear and immediate representation of the risk of bias across the included studies (Figure 2).

### Statistical Analysis

To find out the difference in the magnitude of sensory recovery, a meta-analysis was conducted to pool the effect sizes (mean differences) from individual studies. Given the potential for heterogeneity across studies, a random-effects model was employed using the DerSimonian and Laird method. This model assumes that the true effect size varies across studies and follows a normal distribution. Subgroup analysis was performed to separately evaluate the effect sizes for the anterior and lateral groups. Given the anticipated heterogeneity among the included studies, this analysis was pivotal. The expected heterogeneity stemmed from variations in study populations, interventions, and outcome measures. Study populations might differ in demographics and baseline conditions. Interventional differences could include variations in surgical techniques and the skill level of surgeons. Finally, outcome measures might vary in terms of the tools used to assess sensory recovery, all contributing to the heterogeneity in effect sizes. The heterogeneity among studies within each subgroup and between subgroups was assessed using the *I*^2^ statistic. This statistic describes the percentage of total variation across studies that is due to heterogeneity rather than chance, with *I*^2^ values of 25%, 50%, and 75% indicating low, moderate, and high heterogeneity, respectively. Such quantification allowed us to address the complexity and variability of the data, thereby ensuring a comprehensive synthesis of the available evidence. All statistical analyses were conducted using R (Version 4.3.2), with the “meta” and “metafor” packages facilitating the meta-analysis, and “ggplot2” utilized for the generation of visualizations.

## 3. Results

We identified five studies for the anterior group (Cornelissen et al., 2018 [33]; Beugels et al., 2019 [39]; Bijkerk et al., 2020 [41]; Beugels et al., 2021 [27]; Bijkerk et al., 2022 [40]) and five studies for the lateral group (Yap et al., 2005 [34]; Temple et al., 2006 [44]; Temple et al., 2009 [45]; Mori et al., 2011 [43]; Magarakis et al., 2013 [42]).

### 3.1. General Assessment of Included Studies

The majority of the studies are of Level II evidence and are prospective in nature, with all indicating a consecutive series. While the anterior group predominantly utilized the DIEP method for reconstruction, the lateral group mainly adopted the TRAM method, except for one study. In terms of quality-of-life assessment, only a few studies specified their tools, with BREAST-Q being the most common in the anterior group and the Functional Assessment of Cancer Therapy-Breast QOL instrument used once in the lateral group. Complications related to neurotization were either unreported or indicated no complications in a few studies. Notably, all studies met the criteria for inclusion based on the Joanna Briggs Institute (JBI) checklist (Table 1).

### 3.2. Comparison of Patient Demographics and Neurotization Characteristics

In the studies related to breast reconstruction, the anterior group had a total of 225 non-neurotized and 240 neurotized breasts, while the lateral group consisted of 62 non-neurotized and 51 neurotized breasts. In Mori et al.’s study, inclusion was restricted to cases from skin-sparing mastectomies due to non-comparability of non-innervated control values in the sensory test from other mastectomy types [43]. The predominant method of neurotization across both groups was end-to-end coaptation. However, several studies in the anterior group, including Cornelissen et al., 2018 [33], Beugels et al., 2019 [39] and Bijkerk et al., 2020 [41], added glue to this method for enhanced coaptation. When it came to sensory testing, the Semmes–Weinstein monofilament emerged as the most commonly used method across both groups. However, two studies, one from each group (Bijkerk et al. 2022 [40] in Anterior and Temple et al. 2009 [45] in Lateral), did not conduct any sensory testing. Unique to the lateral group, Magarakis et al. employed the Pressure-Specified Sensory Device for their testing. To compare with monofilament tests, average static sensation values were substituted with corresponding monofilament values [42]. In terms of the number of areas tested for sensory perception, the majority of studies in the anterior group focused on nine areas. In contrast, the lateral group displayed more variation, with tests spanning from 6 to 13 areas, depending on the study (Table 2).

### 3.3. Comparison of Magnitude of Sensory Recovery

**Figure 3:**
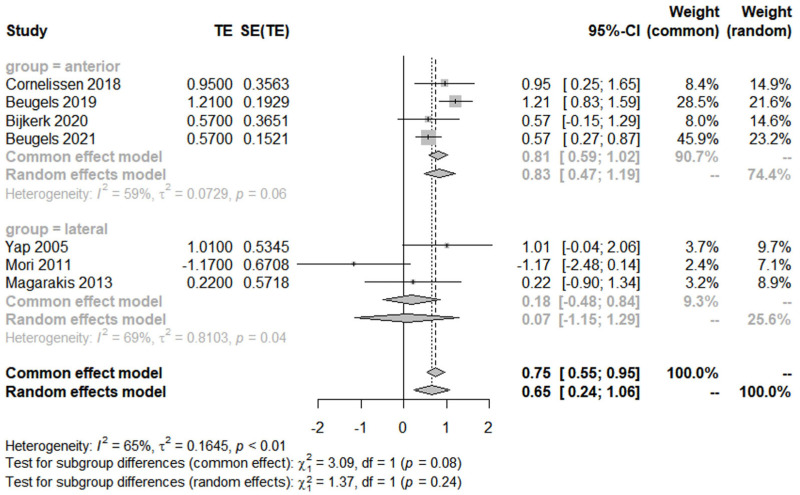
Meta-analysis of mean differences between anterior and lateral groups across studies [27,33,34,39,40,41,42,43,44,45].

The forest plot visually represents the mean difference from each study as well as the pooled mean difference for both subgroups (Figure 3). Each study’s effect size is represented by a square, with the size of the square being proportional to the study’s weight in the meta-analysis. Horizontal lines through the squares represent the 95% confidence intervals (CIs) for each study’s effect size. The pooled effect size for each subgroup is represented by a diamond, with the width of the diamond representing the 95% CI for the pooled effect size. The *I*^2^ statistic indicated moderate to high heterogeneity within the “anterior” group and low heterogeneity within the “lateral” group. This suggests that the variation in effect sizes within the “anterior” group might be due to factors other than chance. The test for subgroup differences was conducted to determine if the pooled effect sizes for the “anterior” and “lateral” groups were statistically different. The result was borderline significant, suggesting a trend towards a difference between the two groups. The anterior group exhibited superior sensory recovery compared to the lateral groups (*p* = 0.08 for the common effect model).

### 3.4. Comparison of Patient-Reported Outcomes

Cornelissen et al., 2018 [33] and Bijkerk et al., 2022 [40] reported superior patient-reported outcomes in neurotized breasts compared to non-neurotized counterparts within the anterior group. Specifically, both studies utilized the physical well-being of the chest domain from the BREAST-Q Reconstruction Module and observed marked improvements in sensory-related quality of life after neurotization. In contrast, no studies in the lateral group provided comparable patient-reported outcome data, highlighting a notable gap in the current literature.

## 4. Discussion

Breast reconstruction after mastectomy has evolved from a volume-restorative procedure into a multifaceted reconstructive endeavor encompassing aesthetic, functional, and psychosocial dimensions. Among emerging priorities, the restoration of breast sensation—particularly through neurotization—has garnered increasing attention for its potential to enhance long-term quality of life. While this study focuses primarily on the comparative outcomes of anterior and lateral intercostal nerve neurotization, a comprehensive understanding of its clinical relevance warrants contextualization within the broader reconstructive landscape. Therefore, to fully appreciate the implications of neurotization, it is important to first consider recent advancements across implant-based reconstruction, autologous tissue transfer, and fat grafting—each of which offers unique challenges and opportunities with regard to sensory recovery and reconstructive integration.

Within implant-based reconstruction—the most commonly employed modality worldwide—recent innovations have enhanced both aesthetic predictability and biological integration. Recent years have seen increasing technical diversity within this modality, particularly in prepectoral placement strategies [47]. Beyond the general acknowledgment of aesthetic benefits, recent evidence emphasizes the importance of intraoperative decision-making based on mastectomy flap thickness, perfusion characteristics, and patient body habitus. For example, the use of intraoperative indocyanine green (ICG) angiography has become a valuable tool for assessing flap viability, potentially reducing complications such as necrosis and explantation [48]. Moreover, the decision to employ prepectoral versus subpectoral implant positioning increasingly depends on individualized perfusion maps rather than fixed algorithms, allowing for a more tailored reconstructive plan [49]. These developments reflect a broader shift toward precision-based implant reconstruction, in which flap integrity and implant selection are optimized intraoperatively to improve both short- and long-term outcomes.

Another underrecognized advancement in implant-based reconstruction is the emergence of hybrid techniques incorporating autologous fat or dermal matrices not just for structural support but for dynamic tissue modulation. For example, the use of fat grafting around the implant pocket has shown potential in modulating capsular tension and reducing contracture rates, particularly in revision cases or irradiated fields. Furthermore, newer generations of acellular dermal matrices and synthetic meshes differ in their inflammatory profiles, and emerging data suggest that the choice of matrix can significantly influence the immune microenvironment around implants, affecting seroma formation, fibrosis, and even pain [50,51,52]. As such, the implant-based approach has moved beyond a binary volumetric solution to a platform that allows biologically integrated, responsive reconstruction.

In autologous reconstruction, especially abdominal-based flaps such as DIEP and its variants, the refinement of flap design and recipient vessel selection has significantly improved reconstructive reliability. Recent developments have highlighted the role of microvascular algorithms that incorporate perforator dominance, vessel caliber matching, and flow dynamics when planning anastomoses. Additionally, use of robotic assistance and ergonomic microdissection techniques has decreased surgeon fatigue and operative time, particularly in bilateral or stacked flap cases [53]. These technical enhancements, although subtle, contribute to lower partial flap loss rates and improved flap inset accuracy, especially in challenging chest wall geometries.

Fat grafting, traditionally viewed as a contouring adjunct, is now emerging as a regenerative tool with cellular and immunomodulatory implications [54]. Beyond its volume-filling role, studies have reported that adipose-derived stromal cells within grafted fat contribute to tissue neovascularization, dermal thickening, and improved radiated skin quality [55,56]. Notably, irradiated breast beds receiving serial fat grafting demonstrate histologic evidence of reduced fibrosis and vascular dropout, suggesting that fat may play a reparative, rather than merely cosmetic, role [57]. These biological effects are particularly relevant in patients undergoing delayed reconstruction or those with implant failure in irradiated fields. Additionally, the standardization of fat harvesting and processing methods, such as low-speed centrifugation, closed-system filtration, and cell-assisted lipotransfer, has improved both graft retention and safety profiles. These advances address previous concerns about oncologic risk and volume unpredictability, particularly in patients with a history of breast cancer. While further prospective data are warranted, current meta-analyses show no increase in local recurrence or mortality associated with fat grafting and support its use in a broader range of reconstructive scenarios.

In parallel with the evolution of aesthetic and structural goals in breast reconstruction, attention has increasingly turned to the restoration of breast sensation as a critical element of postoperative quality of life. Remarkably, sensory recovery can be partially achieved even without neurotization. A seminal paper from 1996 highlighted that after undergoing a free TRAM flap procedure without neurotization, more than 80% of patients experienced a return of breast sensation [58]. This sensitivity was noted to progressively improve over time, a process that can be described as spontaneous sensory recovery. Questions regarding the anatomical possibility of spontaneous reinnervation were raised, but a 2015 study indirectly confirmed its occurrence by documenting the appearance of herpes along the dermatome in the TRAM flap in cases of delayed free TRAM [59].

Building upon the foundational knowledge of spontaneous recovery, neurotization in DIEP flap breast reconstruction introduces a nuanced layer of sophistication to sensory restoration. The pioneering work by Allen in 1994, which described neurotization in DIEP flaps, represented a significant milestone [20]. This study was notable for its successful neurotization of the fourth intercostal nerve and its sensory branch in their fourth case, setting a precedent for future research. The significance of Allen’s contribution was further underscored by a 1999 retrospective analysis that evaluated 24 DIEP flap procedures with neurotization [32]. This study not only confirmed faster and qualitatively superior recovery of sensation compared to standard DIEP and free TRAM flaps but also provided a comparative analysis of the outcomes. It specifically highlighted the neurotization of the lateral branch of the fourth intercostal nerve, an approach validated by subsequent research that predominantly employed the Semmes–Weinstein monofilament test for sensation assessment. Further investigations, such as a 2013 study that explored the use of nerve grafts, although limited by small sample sizes, suggested that neurotization could significantly improve sensation, particularly in patients who did not receive postoperative radiation [42]. Spiegel et al. further compared direct innervation and nerve conduits, concluding that, direct innervation was better than no innervation, and nerve conduits were even more effective [31].

The landscape of sensory restoration in breast reconstruction was comprehensively reviewed in a 2018 systematic review, incorporating 37 studies that spanned various reconstructive techniques [26]. This review concluded that neurotization facilitates faster and more substantial sensory recovery, although the heterogeneity among the studies presented challenges in forming definitive conclusions. A groundbreaking 2019 prospective study provided visual evidence of neurotization’s efficacy in both immediate and delayed reconstruction settings, showcasing enhanced sensitivity in neurotized DIEP flaps through the Semmes–Weinstein test [39].

The cumulative evidence strongly supports that, while spontaneous nerve recovery can naturally occur to an extent, the deliberate integration of neurotization in abdominal flap breast reconstruction markedly improves sensory recovery. This evolution in surgical techniques underscores the importance of neurotization as a critical component of the reconstructive process, aiming to significantly improve the postsurgical quality of life for patients [28]. The strategic selection of the recipient nerve, whether it be the anterior cutaneous branch or the lateral cutaneous branch of the intercostal nerves, is crucial for the success of neurotization, reflecting a deepened understanding and refinement of reconstruction methodologies for enhanced patient outcomes [24,27].

Recent literature has extensively explored the anterior cutaneous branch. Studies by Cornelissen et al. and Beugels et al. have reported encouraging outcomes with this nerve, denoting enhanced sensory results and patient contentment [27,33,39]. Bijkerk’s subsequent research further corroborated these findings, positing the anterior cutaneous branch as a potentially superior conduit for consistent sensory restoration [40,41]. The anterior branch’s anatomical trajectory, coursing along the third rib before superficially traversing the internal mammary vessels towards the sternum, facilitates its dissection and preparation for nerve coaptation [60]. However, a potential drawback is the intact state of the anterior branch post-mastectomy, implying its transection could further diminish mastectomy skin flap sensation [61].

Conversely, the lateral branch of the fourth intercostal nerve, primarily innervating the NAC, presents its merits. Studies by Yap et al. underscored this nerve’s potential in neurotization [34]. Subsequent research delved deeper, suggesting comparable sensory outcomes, albeit with a more complex surgical procedure [42,43,44,45]. The lateral location of this nerve introduces a secondary microsurgical field, potentially constraining flap mobility. Furthermore, the donor nerve’s length might occasionally fall short of reaching the lateral branch, necessitating an allograft and further restricting flap movement [62].

Direct comparisons between these branches are hampered by methodological disparities across studies. Sensory measurements in some studies are quadrant-based, while others adopt a detailed nine-point breast system. Variability in control presence, sensory modalities, and testing instruments further complicates direct comparisons [62]. Our meta-analysis offers an exhaustive review of contemporary literature concerning the anterior and lateral cutaneous branches of the intercostal nerves in breast reconstruction. Preliminary results suggest a proclivity for superior sensory recovery in the anterior group. This observation is paramount, as sensory recovery transcends mere tactile sensation, influencing patient satisfaction, holistic quality of life, and psychological health. Nevertheless, the decision between these nerve branches should be bespoke, tailored to each patient’s unique requirements and circumstances. Considerations should encompass the mastectomy type, patient anatomy, and envisioned surgical outcomes.

However, our study is not devoid of limitations. The predominant flap modality in the Anterior cohort was the DIEP, whereas the Lateral cohort predominantly employed the TRAM. This differential might introduce a confounding variable, albeit its definitive impact remains speculative. The paucity of numerical data in certain studies, particularly in the Lateral cohort, constrains a holistic comparative analysis. Pertaining to patient satisfaction metrics, a dearth of studies in the Anterior cohort employing the BREAST-Q quality-of-life assessment precludes a robust comparison with the Lateral cohort. Given the salience of patient-reported outcomes in clinical decision-making, there is an exigent need for further empirical investigations in this domain.

The overarching objective remains unequivocal: enhancing the patient’s quality of life. Both nerve options strive for sensation restoration, postoperative pain mitigation, and the optimization of breast reconstruction’s aesthetic and functional outcomes. The decision between the anterior and lateral cutaneous branches should be predicated on individual patient requisites, surgical acumen, and the most recent systematic review and meta-analysis evidence.

## 5. Conclusions

Breast reconstruction has progressively evolved to address not only aesthetic restoration but also the recovery of sensory function, which plays a critical role in patient satisfaction and overall quality of life. Our meta-analysis indicates a modest yet clinically meaningful preference for utilizing the anterior cutaneous branch of the intercostal nerves in neurotization, owing to its favorable anatomical accessibility, consistent coaptation potential, and promising sensory outcomes demonstrated across multiple studies. Nevertheless, both anterior and lateral branches remain anatomically viable, and the optimal choice should be tailored to each patient’s surgical anatomy, reconstructive plan, and individual needs. Future research should aim to reduce heterogeneity across studies by standardizing outcome measures and incorporating long-term sensory and quality-of-life data. Ultimately, the integration of neurotization—regardless of branch selection—represents a significant advancement in reconstructive breast surgery, contributing to a more holistic and patient-centered approach that prioritizes both form and function.

## Figures and Tables

**Table 1:** Quality assessments of included studies.

Group	Study	Year	Level of Evidence	Study Design	Consecutive Series	Reconstruction Type	Quality-of-Life Assessment	Complication Related to Neurotization	JBI Checklist Overall Appraisal
Anterior	[33]	2018	III	Retrospective	Yes	DIEP	BREAST-Q	No report	Include
	[39]	2019	II	Prospective	Yes	DIEP	No report	No complications	Include
	[41]	2020	II	Prospective	Yes	DIEP	No report	No report	Include
	[27]	2021	II	Prospective	Yes	DIEP	No report	No complications	Include
	[40]	2022	II	Prospective	Yes	DIEP	BREAST-Q	No report	Include
Lateral	[34]	2005	II	Prospective	Yes	TRAM	No report	No report	Include
	[44]	2006	II	Prospective	Yes	TRAM	No report	No report	Include
	[45]	2009	II	Prospective	Yes	TRAM	Functional Assessment of Cancer Therapy-Breast QOL instrument	No report	Include
	[43]	2011	III	Retrospective	Yes	TRAM	No report	No report	Include
	[42]	2013	III	Retrospective	Yes	DIEP	No report	No report	Include

DIEP: deep inferior epigastric perforator flap, TRAM: transverse rectus abdominis myocutaneous flap, JBI: The Joanna Briggs Institute critical appraisal checklist for case series.

**Table 2:** Comparison of patient demographics and neurotization characteristics.

Group	Study	Number of Breasts		Mean Age in Years		Recipient	Method of Neurotization	Method of Sensory Testing	Sensory Testing Number of Areas
		Non-Neurotized Control	Neurotized	Non-Neurotized Control	Neurotized				
Anterior	[33]	14	18	47.7 ± 4.7	47.1 ± 9.2	Anterior cutaneous branch of intercostal	End-to-end coaptation + glue	Semmes–Weinstein Monofilament	9
	[39]	61	48	50.0 ± 7.7	50.3 ± 8.9	Anterior cutaneous branch of intercostal	End-to-end coaptation + glue	Semmes–Weinstein Monofilament	9
	[41]	15	15	49 ± 13		Anterior cutaneous branch of intercostal	End-to-end coaptation + glue	Semmes–Weinstein Monofilament	9
	[27]	80	94	55.2 ± 8.5	52.0 ± 10.3	Anterior cutaneous branch of intercostal	End-to-end coaptation	Semmes–Weinstein Monofilament	9
	[40]	55	65	54 ± 7	52 ± 10	Anterior cutaneous branch of intercostal	End-to-end coaptation	No testing	-
Lateral	[34]	7	7	51 (44–58)	46 (44–51)	Lateral cutaneous branch of intercostal	End-to-end coaptation	Semmes–Weinstein Monofilament	13
	[44]	19	18	52 ± 9.45	55 ± 9.04	Lateral cutaneous branch of intercostal	End-to-end coaptation	Semmes–Weinstein Monofilament	9
	[45]	18	18	51.6	55.1	Lateral cutaneous branch of intercostal	End-to-end coaptation	No testing	-
	[43]	5	4	50.6 (38–60)	41.3 (31–49)	Lateral cutaneous branch of intercostal	End-to-end coaptation	Semmes–Weinstein Monofilament	6
	[42]	13	4	53 ± 10	-	Lateral cutaneous branch of intercostal	End-to-end coaptation	Pressure-Specified Sensory Device	9
